# Exclusive breastfeeding abandonment in adolescent mothers: a cohort study within health primary services*

**DOI:** 10.1590/1518-8345.6252.3786

**Published:** 2022-11-07

**Authors:** María Isabel Nuñez Hernández, Maria Luiza Riesco

**Affiliations:** 1 Universidad de los Andes, Facultad de Enfermería y Obstetricia, Santiago, Chile.; 2 Universidade de São Paulo, Escola de Enfermagem, São Paulo, SP, Brazil.

**Keywords:** Breast Feeding, Adolescent, Health Promotion, Nursing, Obstetric Nursing, Cohort Studies

## Abstract

**Objective:**

to analyze the factors associated with the abandonment of exclusive breastfeeding in adolescent mothers during the first 6 months of the infant’s life.

**Method:**

this is a cohort study of 105 adolescent mothers followed at the child’s 2-, 4- and 6-months of age. The epidemiological approach was adopted, supported by the positivism paradigm. Exposure variables were those directly related to breastfeeding and sociodemographic, family, maternal and child conditions. Data were collected by interview and analyzed by bivariate and multivariate statistics. The Hazard Ratio (HR) was calculated with a 95% confidence interval (95%CI). The tests were performed, admitting an error type I of 5%. The confidentiality of data was ensured.

**Results:**

the cumulative incidences of exclusive breastfeeding abandonment were 33.3%, 52.2% and 63.8%, at 2, 4 and 6 months, respectively. The variables that remain in the final multivariate model were maternal perception of milk quality (HR=11.6; 95%CI 3.6-37.5), pacifier use (HR=1.9; 95%CI 1.2-3.3), and time of first breastfeeding session (HR=1.4; 95%CI 0.5-12.9).

**Conclusion:**

the highest abandonment rate occurs before the fourth month. A perception of having poor-quality milk by the adolescent mother and pacifier use are factors that favor the abandonment of exclusive breastfeeding. Determining the factors associated with breastfeeding abandonment may allow their timely management, especially in more vulnerable populations.

## Introduction

In the last three decades, Chile made sustained progress in its human development based on a balanced combination of economic growth and public policies; however, the country presents growing inequality and a 14.4 poverty rate^1^. Teenage pregnancy is a social and health indicator of this inequality^2^.

The rate of teen pregnancy in Chile has had a variable and worrying progression. According to official statistics, it increased consistently from 1990 to 2008 and then decreased slightly from that year until 2018^3^ ^-^ ^4^. This trend generates a permanent and sustained risk condition since numerous studies agree that an adolescent mother’s newborn child is more physically, psychologically and socially vulnerable, has a lower weight and is more prone to child abuse^4^ ^-^ ^5^.

The World Health Organization (WHO) recommends that mothers around the world exclusively breastfeed (EB) their babies for the first six months of life to achieve optimal growth, development, and health status. On a global scale, EB rates have been fluctuating, generating the global health target “Increase the rate of exclusive breastfeeding in the first six months of life to at least 50%”^6^.

In Chile, EB during the first 6 months of an infant’s life has been established as a health goal: to achieve EB for 60% of infants up to 6 months of age among the population attending community health centers (primary care). Currently, the prevalence of EB is far below the goals proposed by the Ministry of Health, possibly affecting the growth and optimal development of children belonging to more vulnerable populations, such as those of teenage mothers^1^ ^,^ ^7^.

Determining the factors associated with EB abandonment in adolescent mothers will allow for the timely management of these risk factors and favor better child growth and development, especially in more vulnerable populations^8^.

Therefore, the objective of this study was to analyze the factors associated with the abandonment of EB in adolescent mothers during the first 6 months of the infant’s life.

## Method

The epidemiological approach was adopted, supported by the positivism paradigm.

### Design

This is a prospective cohort study.

### Setting

The study was conducted at the seven family health centers (*Centros de Salud Familiar*-CESFAM) in the San Bernardo Commune, located to the south of the Metropolitan Area of Santiago, Maipo Region of Chile^9^.

In 2013, when this study was planned, San Bernardo had an estimated population of 319,517 people, of which 51% were female. The urban population percentage was 98.1%, with a birth rate of 16.3 *per* 1,000 people and infant mortality of 9.9 *per* 1,000 live births. The projected population density is 2,216 inhabitants *per* km^2^ for 2020. The current number of adolescent pregnancies in the commune was 1.2%, higher than the percentage of the whole country (0.9%) and the southern Metropolitan Area (1%). Trends among 15-19 years teenagers (25.6%) are increasing, with figures higher than the country (22.2%) and the Southern Metropolitan Area (23.9%). Nowadays, the number of teenage pregnancies is decreasing in the rest of the country, but remains a significant public health problem in the study setting^10^.

### Sample

The sample was calculated based on EB prevalence in 2013 between the first and sixth month of infants’ lives in the Commune of San Bernardo. The Health Corporation of the Municipality of San Bernardo reports that 76.4% of mothers breastfed their children in the first month of life, and by the sixth month of life, this prevalence drops to 48.6%^10^.

For estimation, the criterion of marginal homogeneity was used according to the McNemar test, which compares paired proportions with α = 5% and a power of 80%. The resulting minimum number of mothers and infants who should be included in the study was 105.

The sample was stratified according to the number of evaluations of healthy children at each health center. For this stratification, the estimated proportion of adolescent mothers in the district of San Bernardo (17.2%)^10^ and the number of children seen for one-month child well-being evaluations were considered.

Adolescent mothers who met the following criteria were included in the cohort: age between 15 and 19.5 years at the time of delivery, non-twin child, medical discharge from the maternity ward along with the infant, no difficulties with the infant’s maternal latch due to illness or a fetal malformation, EB at time of discharge from the maternity ward, capable of understanding what was asked and free of communication difficulties.

The participants were enrolled by convenience when taking the two-month infant for consultation. The researcher was on different days in each health center, and the adolescent mothers who arrived were asked about the inclusion criteria. When the sample size was complete, the enrolment process was stopped.

### Data collection

Firstly, the adolescent mothers were invited to participate and signed an informed consent form.

The participants who attended the 2-month child well-being evaluation (between 55 and 65 days of life) and met the inclusion criteria were included in the cohort. Mothers who continued with EB at 4 months (between 115 and 125 days) and 6 months (between 175 and 185 days) of the infant’s life were followed.

Data were collected from October 2014 to March 2016 via an interview conducted by one of the researchers using a questionnaire. This questionnaire was previously tested (with 10 mothers not included in the cohort) to confirm the adolescent’s understanding of the questions. It considered all the variables investigated and was used for each study stage (2, 4 and 6 months). The questionnaire had two parts: one about general antecedents that were requested once because they did not vary in time (for example, type of delivery), and the other that was adopted during the follow-up. This instrument was developed using based on a literature review, considering the variables affecting breastfeeding abandonment.

### Measurements

The exposure variables were: return to school (continuity of studies after delivery); living with the mother or mother-in-law (at the moment of the interview); stable partner (away from the partner during pregnancy and after delivery); pain when breastfeeding (any time); cracks in the nipple (any time); maternal perception of milk quality (as good, regular or bad, considering her opinion) and maternal perception of the child’s satisfaction when breastfeeding (considering her opinion); receiving education on breastfeeding during the pregnancy and postpartum (from a health professional or someone else, individually or in groups); cesarean section; skin-to-skin contact (immediately after childbirth); pacifier use (any time); postpartum consumption of illicit drugs (except tobacco and alcohol); time of first breastfeeding session (how much time had passed since the baby was born until they were given to the mother to offer the breast; the participant who responded “immediately” was considered within the first 15 minutes; those who said that the baby was first cleaned and sheltered or explained they had been delayed were considered in the first 30 minutes; those who said that it occurred later in the recovery room were considered more than 1 hour); hospitalization of the newborn (after discharge).

The abandonment of EB and the infant’s age at the time of EB abandonment were considered outcomes. EB was considered if the infant only receives breast milk without any additional food or drink, not even water.

### Data analysis

The consistency analysis of data was made to each variable by identifying codes not allowed for the field. Inconsistencies were corrected using the information contained in the instrument.

Descriptive analyses of the variables were performed using frequency and percentage analyses. The accumulated incidences of EB abandonment for each month of follow-up (2, 4 and 6 months) were calculated.

Values between the variables were compared using the log-rank test. This comparison was made to evaluate the survival differences according to the categorical variables that were theoretically interesting to explore regarding the EB status over time. Survival curves were obtained using the Kaplan-Meier estimator.

All the variables that presented p-values ≤ 0.10 were considered for constructing the multivariate model. The Cox proportional hazards survival model^11^ was adopted for the multivariate analysis of EB abandonment. The risk ratio was calculated using the hazard ratio (HR) with 95% confidence intervals (95%CI). The “forward stepwise” input method was applied to the variables considered in the construction of the model to detect those that were more significant.

The tests were conducted using a type 1 error probability and p ≤ 0.05 was adopted.

### Ethical aspects

The Research Ethics Committee of the School of Nursing at the University of São Paulo, Brazil, and the Scientific Ethics Committee of the South Area of Santiago, Chile, approved this study. 

Mothers’ participation was voluntary and followed all the determinations, ensuring the protection of the rights of all involved in the research.

The measures taken to protect data confidentiality included privacy at the location of the interviews, the anonymity of information by coding the participants in the database and the restriction of data access to the researchers.

## Results

In this study, 248 adolescent mothers were evaluated for eligibility in seven CESFAM locations in the Commune of San Bernardo; 125 did not meet the inclusion criteria, and 18 refused to participate. Thus, 105 participants and their 2-month-old children were included in the cohort and followed at 4 months (n=70) and 6 months (n=47) between October 2014 and March 2016.

Regarding the ages of the participants, 51.4% (n=54) were less than 17 years old, 95.2% (n=100) had completed primary education, and 65.7% (n=69) had normal childbirth.

Of 105 (100%) participants, 35 (33.3%) had already abandoned EB when the infants were 2 months old. At 4 months of the 70 (100%) participants who remained in the study, 23 (32.9%) had incorporated another food, and at 6 months, of the 47 (100%) mothers who remained, 9 (19.1%) had added another food. The remaining 38 (80.1%) participants were continuing EB. Thus, the EB dropout rates were 33.3%, 32.9%, and 19.1%, at, respectively, 2, 4 and 6 months.

**Figure 1:**
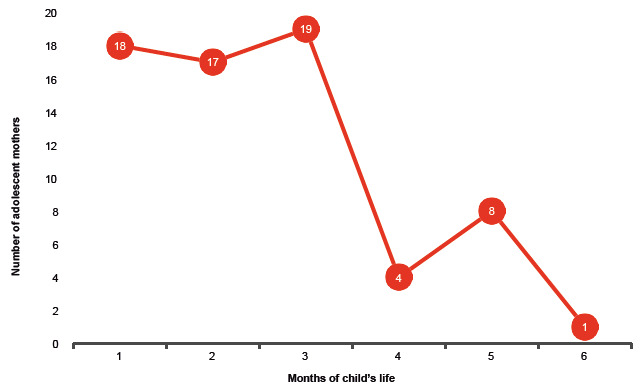
Abandonment of exclusive breastfeeding in adolescent mothers (n=67), month by month, from the first to the sixth month of the child’s life. San Bernardo, Maipo, Chile, 2014-2016

The number of mothers who abandoned EB every month, up to the 6-month evaluation point is presented in Figure 1. The cumulative incidence of EB abandonment was 33.3%, 52.2% and 63.8%, at 2, 4 and 6 months, respectively. It is worth noting that the information regarding EB at the first, third and fifth months of life was obtained retrospectively.

The bivariate analysis of the exposure variables and the information regarding abandonment at 2, 4 and 6 months is presented in Table 1.

**Table 1:** Distribution of adolescent mothers at 2 (n=105), 4 (n=70) and 6 (n=47) months of the child’s life, according to the exclusive breastfeeding, factors related to the abandonment, and p-value. San Bernardo, Maipo, Chile, 2014-2016

Variable	Abandonment of the EB									
	2 months			4 months			6 months			p-value*
	Yes n (%)	No n (%)	Total n (%)	Yes n (%)	No n (%)	Total n (%)	Yes n (%)	No n (%)	Total n (%)	
Return to school										0.004*
Yes	10 (9.5)	2 (1.9)	12 (11.4)	6 (8.6)	2 (2.9)	8 (11.4)	5 (10.6)	10 (26.3)	15 (31.9)	
No	25 (23.8)	68 (64.8)	93 (88.6)	17 (24.3)	45 (62.2)	62 (88.6)	4 (8.5)	28 (59.6)	32 (68.1)	
Living with the mother or mother-in-la										0.112
Yes	27 (25.7)	44 (41.9)	71 (67.6)	13 (18.5)	28 (40.1)	41 (58.6)	6 (12.8)	28 (59.5)	34 (72.3)	
No	8 (7.6)	26 (24.8)	34 (32.4)	10 (14.3)	19 (27.1)	29 (41.4)	3 (6.4)	10 (21.3)	13 (27.7)	
Stable partner										0.780
Yes	25 (23.8)	60 (57.2)	85 (81.0)	23 (32.9)	38 (54.2)	61 (87.1)	8 (17.0)	27 (57.5)	35 (74.6)	
No	10 (9.5)	10 (9.5)	20 (19.0)	-	9 (12.9)	9 (12.9)	1 (2.1)	11 (23.4)	12 (25.5)	
Breast pain										0.969
Yes	12 (11.4)	18 (17.1)	30 (28.6)	5 (7.1)	11 (15.7)	16 (22.9)	-	12 (25.5)	12 (25.5)	
No	23 (21.9)	52 (49.6)	75 (71.4)	18 (25.7)	36 (51.5)	54 (77.1)	9 (19.1)	26 (55.4)	35 (74.5)	
Cracks in the nipple										0.045*
Si	23 (21.9)	36 (34.3)	59 (56.2)	8 (11.4)	15 (21.4)	23 (32.9)	1 (2.1)	9 (19.1)	10 (21.3)	
No	12 (11.4)	34 (32.4)	46 (43.8)	15 (21.4)	32 (45.8)	47 (67.1)	8 (17.0)	29 (61.8)	37 (78.7)	
Perception of milk quality										<0.001*
Good	3 (2.9)	59 (56.1)	62 (59.0)	5 (7.1)	43 (61.4)	48 (68.6)	4 (8.5)	35 (74.5)	39 (83.0)	
Regular or bad	32 (30.5)	11 (10.5)	43 (41.0)	18 (25.7)	4 (5.7)	22 (31.4)	5 (10.6)	3 (6.4)	8 (17.0)	
Perception child satisfactions										<0.001*
Yes	9 (8.6)	63 (59.9)	72 (68.6)	6 (8.6)	38 (54.3)	44 (62.9)	4 (8.5)	3 (6.4)	7 (14.9)	
No	26 (24.8)	7 (6.7)	33 (31.4)	17 (24.3)	9 (12.9)	26 (37.1)	5 (10.6)	35 (74.5)	40 (85.1)	
Education for breastfeeding during pregnancy										0.497
Yes	26 (24.8)	54 (51.4)	80 (76.2)	31(73.8)	23(82.1)	54 (77.1)	25(80.6)	14(87.5)	39 (83.0)	
No	9 (8.6)	16 (15.2)	25 (23.8)	11(26.2)	5(17.9)	16 (22.9)	6(19.4)	2(12.5)	8 (17.0)	
Education during puerperium										0.945
Yes	28 (26.7)	54 (51.4)	82 (78.1)	34(80.9)	20(71.4)	54 (77.1)	28(90.3)	12(75.0)	40 (85.1)	
No	7 (6.7)	16 (15.2)	23 (21.9)	8(19.1)	8(28.6)	16 (22.9)	3(9.7)	4(25.0)	7 (14.9)	
Cesarean										0.055
Yes	15 (14.3)	13 (12.4)	28 (26.7)	15(35.7)	6(21.4)	21 (30.0)	14(45.2)	4(25.0)	18 (38.3)	
No	20 (19.0)	57 (54.3)	77 (73.3)	27(64.3)	22(78.6)	49 (70.0)	17(54.8)	12(75.0)	29 (61.7)	
Skin-to-skin contact										0.880
Yes	29 (27.6)	59 (56.2)	88 (83.8)	14 (20.0)	24 (34.3)	38 (54.3)	5 (10.6)	14 (29.8)	19 (40.4)	
No	6 (5.7)	11 (10.5)	17 (16.2)	28 (40.0)	4 (5.7)	32 (45.7)	26 (55.3)	2 (4.3)	28 (59.6)	
Pacifier user										<0.001*
Yes	25 (23.8)	16 (15.2)	41 (39.0)	19 (63.3)	4 (10.0)	23 (32.9)	5 (10.6)	2 (4.6)	7 (14.9)	
No	10 (9.5)	54 (51.4)	64 (61.0)	11 (36.7)	36 (90.0)	47 (67.1)	4 (8.5)	36 (76.6)	40 (85.1)	
Use of illicit drugs										<0.001*
Yes	6 (5.7)	-	6 (5.7)	-	-	-	-	-	-	
No	29 (27.6)	70 (66.7)	99 (94.3)	23 (32.9)	47 (67.1)	70 (100)	9 (19.1)	38 (80.9)	47 (100)	
Time of first breastfeeding session (min)										0.032*
< 15 min	1 (0.9)	10 (9.5)	11 (10.5)	3 (4.3)	4 (5.7)	7 (10.0)	2 (4.3)	2 (4.3)	4 (8.5)	
>15 min	34 (32.4)	60 (57.2)	94 (89.5)	39 (55.7)	24 (34.3)	63 (90.0)	29 (61.6)	14 (29.8)	43 (91.5)	
Hospitalization										0.057
Yes	11 (10.5)	13 (12.4)	24 (22.9)	4 (5.7)	9 (12.9)	13 (18.6)	-	3 (6.4)	3 (6.4)	
No	24 (22.9)	57 (54.3)	81 (77.1)	19 (27.1)	38 (54.3)	57 (81.4)	9 (19.1)	35 (74.5)	44 (93.6)	
Total	35 (33.3)	70 (66.7)	105 (100)	23 (32.9)	47 (67.1)	70 (100)	9 (19.1)	38 (80.9)	47 (100)	

*Log-rank test

Variables with statistically significant values included return to school (p=0.004), cracks in the nipple (p=0.045), maternal perception of milk quality (p<0.001), maternal perception of the child’s satisfaction when breastfeeding (p<0.001), pacifier use (p<0.001), postpartum consumption of illicit drugs (p<0.001), and time of first breastfeeding session (p=0.032). Variables with p < 0.10 were tested in the multivariate analysis. Given its p-value in the bivariate analysis, cesarean section and child hospitalization were included (Table 1).

As shown in Table 2, using the “forward stepwise” input method, the following variables were significant in the final model: maternal perception of milk quality (as good, regular, or bad), pacifier use (yes or no) and time of first breastfeeding session (≤15 or >15 minutes after childbirth). If the participants perceived their milk as regular or bad quality, the risk of EB abandonment was 11.6 times higher; if a pacifier was used, it was 1.9 times higher; and if the first breastfeeding session was more than 15 minutes after the birth, it was 1.4 times higher (in this case, the confidence interval was less than 1 and, therefore, can be considered a risk, although not significant).

**Table 2:** Accumulated risk of exclusive breastfeeding abandonment (n=105). San Bernardo, Maipo, Chile, 2014-2016

Variable	HR*	95%CI^†^
Maternal perception of milk quality	11.6	[3.6-37.5]
Pacifier user	1.9	[1.2-3.3]
Time of first breastfeeding session	1.4	[0.5-12.9]

*HR = Hazard ratio; ^†^95%CI = 95% Confidence interval

**Figure 2:**
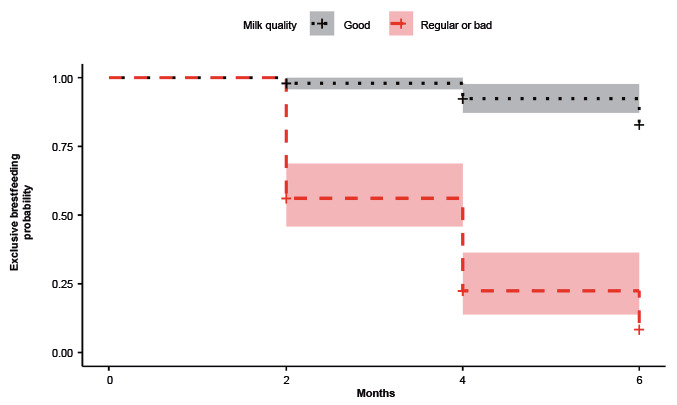
Survival curves and 95% confidence interval of the exclusive breastfeeding according to maternal perception of milk quality (n=105). San Bernardo, Maipo, Chile, 2014-2016

**Figure 3:**
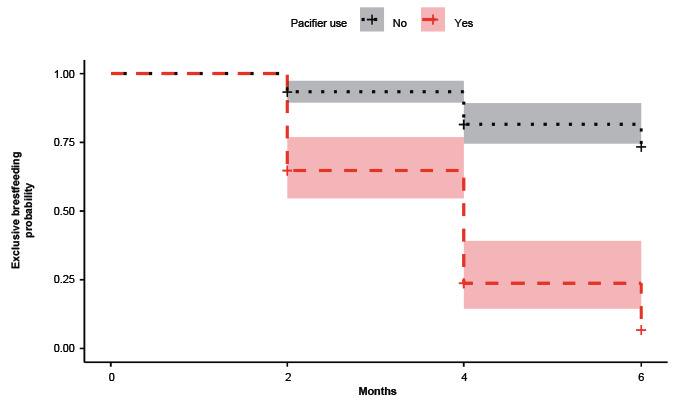
Survival curves and 95% confidence interval of the exclusive breastfeeding according to pacifier use (n=105). San Bernardo, Maipo, Chile, 2014-2016

Next, the EB survival analysis graphs were created for the final model’s significant variables (Figures 2-3). The lines refer to each variable evaluated in a dichotomous way and the 95%CI.

Participants who perceived that their milk was of good quality showed higher EB survival than those who perceived that the quality of their milk as regular or bad (Figure 2). Participants whose children did not use pacifiers showed greater EB survival than those whose children used them (Figure 3).

## Discussion

The present study showed an abandonment rate of EB among participants at 6 months that was greater than the national mean reported by the Ministry of Health (36.2% EB *vs* 43.1%)^12^. However, another study in Chile also found a lower prevalence than those described by the official statistics^13^.

This abandonment rate is consistent with low- and middle-income countries where the EB rate was 37% at 6 months^14^. A study carried out in Mexico found a lower (28%) prevalence of EB at 6 months^15^, as well as studies conducted in Spain and Brazil^16^ ^-^ ^17^. The rate found in Peru was 46.4% at 6 months^18^. In these studies, the age of the mother was not considered.

A study conducted in Ecuador with adolescent mothers determined that 62.9% maintained EB until 6 months of age^19^. A study in Chile found that the mother’s age was correlated with early weaning and that mothers under 26 years of age achieved EB for less time^20^. Other studies found that EB was maintained longer in older mothers^21^ ^-^ ^22^.

Regarding EB abandonment during the first 6 months, the present study shows that the highest abandonment rate occurs in the first 3 months of infant life (52.2% accumulated in the fourth month). This outcome is inconsistent with a study conducted in Spain, in which 53.7% of the cases studied maintained EB until 4 months of age^23^.

Concerning demographic variables, the participants’ ages were mainly between 15 and 16 years old. This outcome is consistent with the maternal demographics of the country, where an increase in adolescent pregnancies at younger ages is found^2^.

The consumption of alcohol, tobacco and illicit drugs (marijuana and cocaine) during pregnancy was lower than the typical rates found among adolescents in Chile^24^. These percentages may be due to a decrease in the consumption of these substances during pregnancy, and we observed a generalized knowledge concerning risks, or the effects of social desirability in the adolescents’ answers. Mothers who admitted alcohol, tobacco and drug use abandoned EB before 4 months of age, which is consistent with a systematic review in which the meta-analysis indicated that mothers who smoked were 2.49 times more likely not to exclusively breastfeed than non-smoking mothers^25^.

Regarding the variables directly associated with EB abandonment in this study, a few participants returned to school at 2 and 4 months (11.4%), whereas a third of those who continued EB returned to school at 6 months. In Chile, school dropouts tend to occur before and during pregnancy, with low reintegration school^26^.

Concerning the mammary difficulties evaluated, cracks in the nipple significantly influenced EB rates, being consistent with the findings of a systematic^27^ and an integrative review^28^, which found that the study population presented cracks in the nipple and also an association between primiparity and previous or current nipple cracks. Likewise, another study confirmed that alterations in the nipples negatively influenced EB^29^.

The maternal perception of breast milk quality and the satisfaction of the infant with EB are two closely related variables that directly involve maternal perceptions, which is consistent with the findings in a longitudinal descriptive study in a Spanish setting^30^. A study conducted in rural Kenya with adolescent mothers corroborates with the effect of these perceptions on breastfeeding^31^.

Pacifiers as a risk factor for EB abandonment has been amply demonstrated^32^. However, it is a practice strongly associated with Chilean culture. Other studies have shown that a mother’s motivation to breastfeed is the most critical factor in maintaining EB. Furthermore, using a pacifier calms the child and prevents sudden infant death syndrome^33^.

The time of first contact, namely, mothers who had contact in the first 15 minutes after birth versus those who had contact after the first 15 minutes, showed a significant relationship with EB. In a recent systematic review and meta-analysis, the quantitative analysis showed that skin-to-skin contact between mother and child had a significantly positive effect on the success of the first lactation and duration of the first lactation^34^.

Other studies in Indonesia determined that an early start to breastfeeding has a statistically significant relationship with EB, where early-onset was considered less than 1 hour after birth^35^ ^-^ ^36^. 

The initiation of physical contact within 15 minutes is related to having a natural childbirth and immediate skin-to-skin contact. The response regarding timing in this question is based on the mother’s memory and perception and was considered to have occurred immediately when it took place within 15 minutes.

A cesarean delivery resulted in a tendentially significant relationship with the abandonment of EB, consistent with what was found in Spain, where cesarean sections were associated with a lower likelihood of EB^37^. In Ethiopia, mothers who had vaginal deliveries were more likely to breastfeed exclusively than those who had a cesarean section^38^. In Chile, a study showed that vaginal delivery was positively associated with EB^39^.

Regarding this study’s limitations, the possibility of bias and the restricted generalization power should be considered.

Regarding the selection bias, it refers mainly to the mode of including the participants, which was for convenience. About implementation, limitations in data collection (performed at the health service, which may constrain the participant) were minimized by using a private room for the interview and because a single researcher, who is not part of the health team, collected all data. Nevertheless, the information bias may be present when the participant provided a socially expected response or wanted to please the researcher (e.g., pacifier use, drug use). Concerning the interpretation of the results, the confounding bias was minimized by using multivariate analysis. In turn, even if the sample had been estimated with adequate parameters, the reduced number of participants at 6 months of infant life-limited an optimal statistical power.

As for the generalization of the results, although teenage pregnancy rates are similar in the different urban regions of Chile, San Bernardo’s reality and life conditions are worse (e.g., higher rates of school dropout and poverty, and lower per capita index) than in other sectors of the metropolitan region, where living conditions are better, with more excellent resources. Despite these, we consider it possible to generalize the main results.

Although the findings of this cohort are relevant, an interval shorter than 2 months to follow the abandonment of EB and the participation of adolescent mothers with diverse social characteristics could provide accurate data on the duration of EB in the first 6 months.

This study advances the understanding, by nurses and other health professionals, of the abandonment of breastfeeding in the most vulnerable populations. By achieving more excellent knowledge about the causes that affect abandonment, we will be able to generate strategies that allow a greater prevalence of exclusive breastfeeding.

## Conclusion

As determined in this study, the EB abandonment rate for adolescent mothers at 6 months is higher than the national EB abandonment rate in Chile. The highest abandonment rate occurs before the fourth month. Therefore, EB promotion strategies must be adopted from the beginning of the pregnancy and, specifically, during the first three months of the infant’s life.

Of the group of variables related to the abandonment of EB that were considered, only two were confirmed among the participants in this cohort.

The perception of milk quality and pacifier use are two variables that can be addressed through education during pregnancy. The type of education delivered is essential, but the transmission of knowledge and not just providing information should be the aim.

The moment of first physical contact between the mother and child is vital because it is associated with a more natural birth centered on the woman and her child but is not on the routine procedures that become a priority in many cases. 
